# A Novel Pallet Detection Method for Automated Guided Vehicles Based on Point Cloud Data

**DOI:** 10.3390/s22208019

**Published:** 2022-10-20

**Authors:** Yiping Shao, Zhengshuai Fan, Baochang Zhu, Minlong Zhou, Zhihui Chen, Jiansha Lu

**Affiliations:** 1 College of Mechanical Engineering, Zhejiang University of Technology, Hangzhou 310023, China; 2 Noblelift Intelligent Equipment Co., Ltd., Huzhou 313100, China

**Keywords:** point cloud data, pallet detection, 3D vision sensor, object recognition, automated guided vehicles

## Abstract

Automated guided vehicles are widely used in warehousing environments for automated pallet handling, which is one of the fundamental parts to construct intelligent logistics systems. Pallet detection is a critical technology for automated guided vehicles, which directly affects production efficiency. A novel pallet detection method for automated guided vehicles based on point cloud data is proposed, which consists of five modules including point cloud preprocessing, key point extraction, feature description, surface matching and point cloud registration. The proposed method combines the color with the geometric features of the pallet point cloud and constructs a new Adaptive Color Fast Point Feature Histogram (ACFPFH) feature descriptor by selecting the optimal neighborhood adaptively. In addition, a new surface matching method called the Bidirectional Nearest Neighbor Distance Ratio-Approximate Congruent Triangle Neighborhood (BNNDR-ACTN) is proposed. The proposed method overcomes the problems of current methods such as low efficiency, poor robustness, random parameter selection, and being time-consuming. To verify the performance, the proposed method is compared with the traditional and modified Iterative Closest Point (ICP) methods in two real-world cases. The results show that the Root Mean Square Error (RMSE) is reduced to 0.009 and the running time is reduced to 0.989 s, which demonstrates that the proposed method has faster registration speed while maintaining higher registration accuracy.

## 1. Introduction

**Figure 1:**
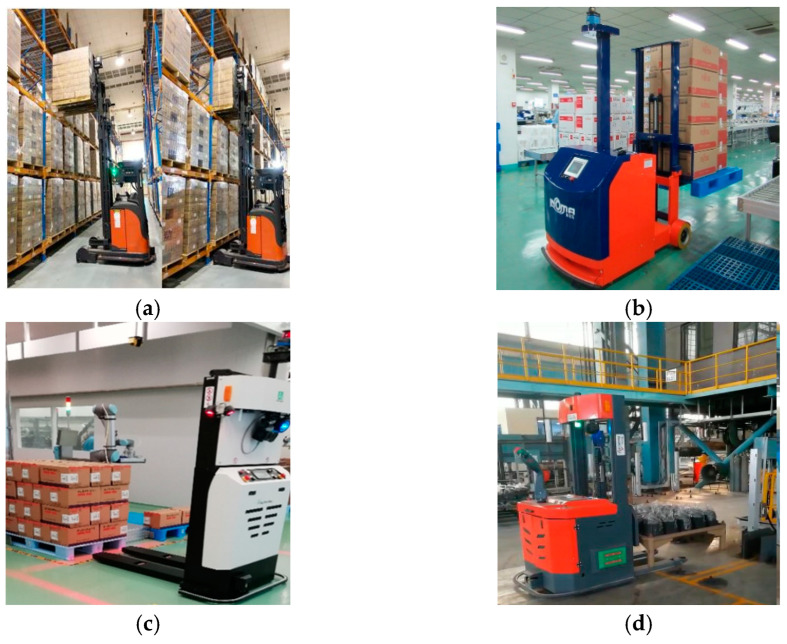
The specific pallet detection scene for automated guided vehicles. (**a**) Storage shelves, (**b**) the production workshop, (**c**) drug transport and (**d**) blast furnace conditions.

Under the background of “Industry 4.0”, the logistics industry is facing challenges, including structural adjustment, industrial optimization, cost reduction and efficiency improvement, and also has ushered in development opportunities such as information technology, intelligent logistics and machine vision [1]. As an important part of an intelligent logistics system, automated guided vehicles are widely used in warehousing, production, service, aerial work and other scenarios, which can establish a human–machine friendly interactive environment and reduce the incidence of safety accidents [2]. However, in the actual storage environment, due to the influence of many factors such as excessive obstacles, uneven illumination, accumulated handling errors and manual intervention, automated guided vehicles have problems of low efficiency and repeated handling in the process of pallet handling [3]. With the help of a 3D vision sensor, automated guidance vehicles can detect the scene pallet, which can effectively solve these problems. Pallet detection for automated guided vehicles is widely used in various scenarios including storage shelves, the production workshop, drug transport and blast furnace conditions, which are shown in Figure 1. The applications of automated guided vehicles in these scenarios can establish a human–machine friendly interactive environment, improve production efficiency and reduce the incidence of safety accidents.

The existing vision-based object detection methods are mainly divided into two categories: the image-based method [4] and the point cloud-based method [5,6]. There has been a large amount of research on the object detection method based on images [7,8,9,10,11]. Specific to pallet detection, Li et al. [12] applied the Region Growing algorithm to extract the whole target region, and the pallet was located by the Progressive Probabilistic Hough Transform (PPHT) method, which solved the problem of difficult target detection under unstable light conditions. Syu et al. [13] used the monocular vision system on the forklift and combined the Adaptive Structure Feature (ASF) and Direction Weighted Overlapping (DWO) ratio to detect the pallet, which removes most of the non-stationary background and significantly increases the processing efficiency. Li et al. [14] established the pallet dataset and applied the improved deep learning object detection algorithm to obtain detection results, which improved the efficiency and accuracy of the pallet detection. The above methods of object detection based on 2D images have been intensively investigated, which is currently a relatively mature research area. However, the imaging process of 2D images involves mapping from 3D space to 2D space, which loses a lot of useful information during the mapping process. Therefore, object detection based on 2D images can no longer satisfy the needs of current industrial production.

With the rapid development of low-cost depth sensors, object detection has converted from traditional single point and segment measurement to dense point cloud and full profile measurement [15,16,17]. Compared with 2D images, 3D point cloud data provide more information about color, texture, geometric feature and space distribution [18], which makes pallet detection based on the 3D point cloud an active research topic. Firstly, the methods based on artificial features were attached to the pallets. Seelinger et al. [19] presented a vision-based approach to identify the fiducials which were placed on each pallet, which provides automated guided vehicle systems with the capability of performing pallet detection tasks. Two reflectors were fixed left and right on the short side of the pallet in the study by Lecking [20] to realize pallet detection. Although these artificial features simplify pallet detection, it takes effort to label all of the pallets in advance, thereby identifying the above approaches as unfeasible. Guo et al. [21] summarized the existing local feature detection methods and concluded that the contradiction between descriptiveness and computational efficiency of the local feature descriptor was a major challenge faced in feature extraction. Hence, it was essential to construct a robust and descriptive feature descriptor. The Fast Point Feature Histogram (FPFH) is a commonly used local feature descriptor which can perform well in descriptiveness, robustness and efficiency [22]. Additionally, FPFH employed the geometric feature of the pallet to build a descriptor without adding any artificial marks. Tao et al. [23] combined SVM classification and the FPFH descriptor to achieve object detection, which improved the robot detection ability and perception in three-dimensional space. A new point registration algorithm that combines FPFH and greedy projection triangulation was presented by Liu et al. [24], which improved the accuracy of registration. Li et al. [25] proposed a novel method of point registration called the Four Initial Point Pairs (FIPP) algorithm based on the FPFH feature descriptor, and the accuracy of FIPP could reach a better level, but it had low efficiency in mass data. However, few studies considered the color information and the criteria for the selection of the neighborhood radius in the FPFH descriptor. Most researchers adjusted the neighborhood radius manually based on prior knowledge, with certain randomness, low efficiency and high complexity.

In response to the above problems, a novel pallet detection method for automated guided vehicles based on point cloud data is proposed, including point cloud preprocessing, key point extraction, feature description, surface matching and point cloud registration. The main contributions can be summarized as: (1) the proposed method considers the HSV color feature, which improves the detection accuracy; (2) an ACFPFH feature descriptor is proposed and the criteria for adaptive selection of the optimal neighborhood radius are established; (3) a new surface matching method called the Bidirectional Nearest Neighbor Distance Ratio-Approximate Congruent Triangle Neighborhood (BNNDR-ACTN) is proposed, which increases the efficiency and accuracy. The proposed method not only overcomes the drawback of randomness and inefficiency of neighborhood selection in traditional feature extraction but also improves the accuracy and efficiency of pallet detection. Moreover, the proposed method can be well adapted to a variety of complex scenes such as the ground and the shelf.

The rest of the paper is organized as follows: In Section 2, the proposed pallet detection method based on the ACFPFH feature descriptor is described. Section 3 outlines two specific case studies and further comparison analysis for verifying the proposed method in engineering applications. Finally, Section 4 concludes this paper.

## 2. The Proposed Method

### 2.1. Overview of the Proposed Method

**Figure 2:**
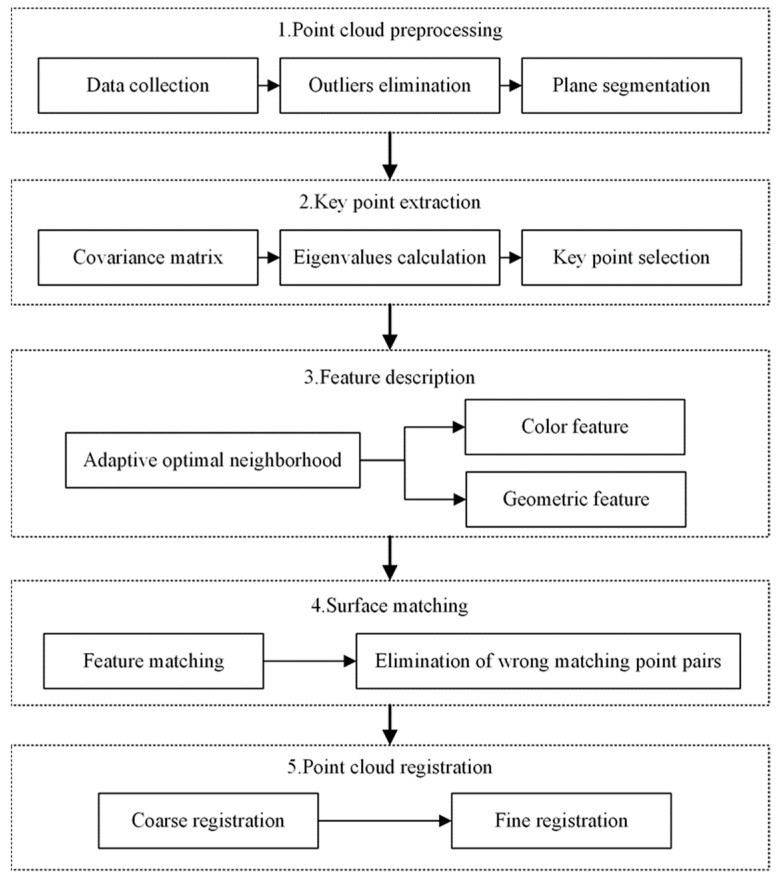
The framework of the proposed approach.

This section describes an overview of the proposed method. It consists of five modules: point cloud preprocessing, key point extraction, feature description, surface matching and point cloud registration. The framework of the proposed method is shown in Figure 2. The procedure involves the following steps.

Step 1: Point cloud preprocessing. The Percipio FM851-E2 3D vision sensor is used to acquire the point cloud data which represent the whole scene, including the pallet. Outliers are eliminated and the redundant information, such as walls and grounds, is removed using Random Sample Consensus (RANSAC) algorithm.

Step 2: Key point detection. The key points with rich information are extracted from scene point clouds by the Intrinsic Shape Signatures (ISS) algorithm.

Step 3: Feature description. The optimal neighborhood radius of each point is obtained based on the minimum criterion of the neighborhood feature entropy function. The color components and the geometric information based the optimal neighborhood of the key point are encoded into a representative feature descriptor called the Adaptive Color Fast Point Feature Histogram. The pallet template point cloud and its corresponding library of feature descriptors are obtained by performing the above three steps.

Step 4: Surface matching. The matching method based on the Bidirectional Nearest Neighbor Distance Ratio (BNNDR) is employed to complete feature matching between the pallet template point cloud and the scene point cloud. Considering that there are some incorrect matching point pairs which will reduce the registration accuracy, it is essential to eliminate them by the Approximate Congruent Triangle Neighborhood (ACTN).

Step 5: Point cloud registration. The RANSAC algorithm is applied for performing point cloud coarse registration, which can obtain the relationship between the template point cloud and the scene point cloud and provide an ideal initial position for fine registration. The fine registration works to obtain a final optimal transformation matrix using the Iterative Closest Point (ICP) algorithm.

### 2.2. Point Cloud Preprocessing

#### 2.2.1. Outliers Elimination

Due to the hardware design of the Percipio FM851-E2 3D vision sensor, external environmental interference and other factors, point cloud outliers are inevitable in the measurement. The pallet detection results will have errors if the outliers in the original scene point cloud Q_so_ are not eliminated.

The distance from arbitrary point $P_{i}$ in the point cloud to its neighborhood point $P_{\mathrm{ik}}(k=1,2,\ldots ,m)$ is approximately subject to Gaussian distribution, and the probability density function of the average neighborhood distance is listed below:

(1) $$f(d_{i})=\frac{1}{\sqrt{2\pi }\sigma }\exp(-\frac{{\left(d_{i}-\mu \right)}^{2}}{2\sigma^{2}})$$

where i=1,2,…,n, n represents the number of points in the point cloud, $d_{i}$ is the average neighborhood distance of arbitrary point $P_{i}$, μ and σ are the expectation and standard deviation of the average neighborhood distance $d_{i}$, respectively. Calculating the average neighborhood distance $d_{i}$, the point $P_{i}$ is considered as an outlier and removed if $\mu -\sigma <d_{i}<\mu +\sigma$.

#### 2.2.2. Plane Segmentation

In warehousing environments, the scene point cloud acquired by the Percipio FM851-E2 3D vision sensor contains a lot of redundant information, such as the grounds and the walls, which will decrease the calculation efficiency. Therefore, it is necessary to remove the useless planes in the scene point cloud [26]. The specific segmentation procedures are as follows:

Step 1: The plane equation in the three-dimensional point cloud is defined as:

(2) Ax+By+Cz+D=0

where A, B and C are plane parameters, and D is the distance from the plane to the point P_i_. Randomly select three points from the scene point cloud Q_SE_ after removing outliers and obtain the parameters of the initial plane P_I_.

Step 2: Calculate the distance D_i_ from the point P_i_ to the initial plane P_I_ and the angle $\beta_{i}$ between the point Pi and the normal vector of the initial plane $P_{I}$. Set distance threshold $D_{\varepsilon }$ and angle threshold $\beta_{\varepsilon }$; if both $D_{i}<D_{\varepsilon }$ and $\beta_{i}<\beta_{\varepsilon }$ are satisfied, the point $P_{i}$ belongs to the plane P_I_.

Step 3: Repeat the above procedures until the number of the points in the plane reaches the threshold t, and remove the final fitted plane model to obtain the preprocessed scene point cloud Q_s_.

### 2.3. Key Point Extraction

The preprocessed scene point cloud Q_s_ still contains a large number of points, which leads to low efficiency of feature extraction and matching. Selecting key points to simplify the point clouds can retain the features of the point clouds as much as possible while reducing the number of the points. The Intrinsic Shape Signatures (ISS) is a widely used algorithm with a fast calculation speed and high repeatability to realize key point extraction [27]. The extraction procedures of the key points PF_i_ are summarized as follows:

Step 1: The neighborhood points $P_{\mathrm{ik}}(k=1,2,\ldots ,m)$ of $P_{i}$ in the scene point cloud $Q_{S}$ are searched within a certain radius d_p_. d_p_ is the average closest point distance of the point cloud collected by the 3D vision sensor, which can be calculated as follows:

(3) $$d_{p}=\frac{1}{N}\sum_{}^{}d_{m}$$

where N is the number of the points, and $d_{m}$ is the distance between each point and its closest point.

Compute a weight parameter $\omega_{\mathrm{ik}}$ for each point $P_{i}$ inversely related to the distance from $P_{\mathrm{ik}}$ to $P_{i}$ as follows:

(4) $$\omega_{\mathrm{ik}}=\frac{1}{\left\|P_{\mathrm{ik}}-P_{i}\right\|}$$

Step 2: The covariance matrix C_i_ of point P_i_ is generated as follows:

(5) $$C_{i}=\frac{\sum_{k=1}^{m}\omega_{\mathrm{ik}}(P_{ik}-\overset{-}{P_{i}}){\left(P_{ik}-\overset{-}{P_{i}}\right)}^{T}}{\sum_{k=1}^{m}\omega_{\mathrm{ik}}}$$

where m is the number of the neighborhood points $P_{\mathrm{ik}}$, and $\overset{-}{P_{i}}$ is the center point of the neighborhood points $P_{\mathrm{ik}}$ and $\overset{-}{P_{i}}=\frac{1}{m}\sum_{k=1}^{m}P_{\mathrm{ik}}$.

Step 3: Calculate the eigenvalues of the covariance matrix C_i_ and sort them from large to small as $\left\{\lambda_{1}^{i},\lambda_{2}^{i},\lambda_{3}^{i}\right\}$.

Step 4: Set the thresholds k_2_ and k_1_; the points with the relation of $\frac{\lambda_{2}}{\lambda_{1}}<k_{1}\cap \frac{\lambda_{3}}{\lambda_{2}}<k_{2}$ are considered as the key point PF_i_.

### 2.4. Feature Description

**Figure 3:**
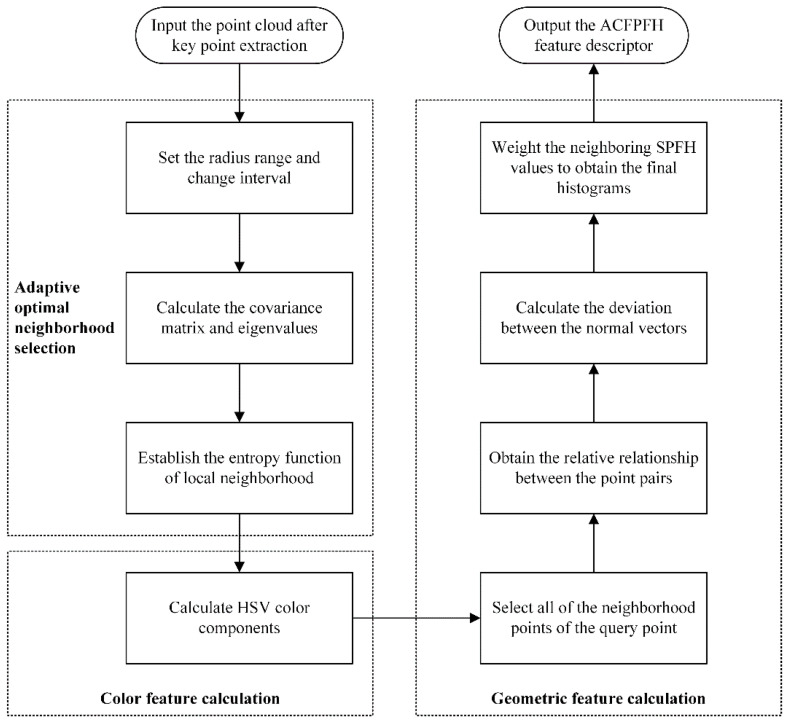
The flowchart of the feature description.

As for the traditional feature descriptors, the neighborhood radius for all the points is a fixed value. Most studies select the appropriate radius based on empirical knowledge, which has strong subjectivity and low efficiency. Besides, the color information is ignored, which makes it difficult to fully and accurately characterize the objects. Therefore, a novel feature descriptor called ACFPFH is defined, which adaptively selects the optimal neighborhood radius and considers the color and geometric features. The flowchart of the proposed feature description method is shown in Figure 3, and the detailed procedures are described as follows.

#### 2.4.1. Adaptive Optimal Neighborhood Selection

The choice is preferred where the radius is more flexible and allowed to vary within a dataset. The proper neighborhood radius obtained by adaptive selection can reduce the runtime of feature extraction under the premise of ensuring precision. Therefore, a general method for obtaining the adaptive optimal neighborhood radius r_opt_ is proposed in this paper, without the limit of prior knowledge. The detailed procedures about depriving the adaptive optimal neighborhood radius r_opt_ are described as follows.

Step 1: Set the radius range [r_min_, r_max_] and change interval Δr of the neighborhood search. Set the value of $r_{\min}$ equal to the average closest point distance d_p_, and $r_{\max}$ is the maximal acceptable neighborhood radius for all the points of the scene point cloud Q_s_, which can usually be set to a fixed value.

Considering the radius of interest is usually closer to $r_{\min}$ than to $r_{\max}$, the radius is calculated as follows:

(6) $$r_{1}=r_{\min},\;r_{j+1}=\left\{ \begin{array}{ll} r_{j}+\Delta r & \mathrm{if}\;r_{j+1}<r_{\mathrm{mid}}\\ r_{j}+2\Delta r & \mathrm{else} \end{array}\right.$$

where $r_{\mathrm{mid}}=\frac{r_{\min}+r_{\max}}{2}$, j=1,2,3,… until $r_{j}>r_{\max}$ and Δr is the adaptive neighborhood radius value step. It results in more reasonable samples near the radius of interest and less when reaching the maximal values.

Step 2: Calculate the covariance matrix C_j_ and eigenvalues $\lambda_{1},\lambda_{2},\lambda_{3}$ of each neighborhood radius $r_{j}$, which can determine the dimensionality characteristics of the local neighborhood. Table 1 shows the details about the dimensionality characteristics [28]. Construct the dimensionality features, including the 1D linearity feature $L_{\lambda }$, 2D planarity feature $P_{\lambda }$ and 3D scattering feature $S_{\lambda }$. They are represented as:

(7) $$L_{\lambda }=\frac{\lambda_{1}-\lambda_{2}}{\lambda_{1}},\;P_{\lambda }=\frac{\lambda_{2}-\lambda_{3}}{\lambda_{1}},\;S_{\lambda }=\frac{\lambda_{3}}{\lambda_{1}}$$

where $L_{\lambda }+P_{\lambda }+S_{\lambda }=1$, and each of them can be regarded as the probability of the point $P_{i}$ being labeled as a 1D, 2D or 3D structure. Consequently, the task of searching for an optimal neighborhood size can be converted to finding which radius favors the corresponding dimensionality.

Step 3: The entropy function of local neighborhood E_neiborhood_ is established as a measure of unpredictability based on information entropy theory, and it is defined as [29]:

(8) $$E_{\mathrm{neighborhood}}=-L_{\lambda }\ln(L_{\lambda })-P_{\lambda }\ln(P_{\lambda })-S_{\lambda }\ln(S_{\lambda })$$

The smaller the value of the information entropy, the smaller the uncertainty of the variable, which is the core of the Shannon entropy theory [30]. Accordingly, it can be concluded that the smaller the information entropy value of the local neighborhood, the less the uncertainty of the dimensional feature of the points. That is, the greater the probability that the point belongs to a certain dimensional feature, and the more similar the spatial distribution characteristics of the local data points under the neighborhood radius, then the neighborhood radius tends to be more optimal. More immediately, it is feasible to obtain the adaptive optimal neighborhood radius $r_{e\text{-}\mathrm{opt}}$ according to the minimum criterion of neighborhood entropy function.

(9) $$r_{e\text{-}\mathrm{opt}}=\mathrm{argmin}(E_{\mathrm{neighborhood}})$$

However, the optimal neighborhood radius $r_{e\text{-}\mathrm{opt}}$ obtained according to Equations (7) and (8) is based on the assumption that obvious dimensionality characteristics exist in the observed point cloud. When the dimensionality features of the point $P_{i}$ are indistinguishable, the optimality of the estimated neighborhood cannot be determined.

In order to avoid the limitation of the above assumptions for the scene point cloud and improve the estimation accuracy of the optimal neighborhood, a more general solution for calculating the optimal neighborhood radius $r_{\mathrm{opt}}$ is proposed in this paper. The eigenvalues directly reflect the dimensional distribution characteristics of the neighborhood points. Consequently, the three eigenvalues are normalized by their sum $\sum \lambda_{j}$ for obtaining an eigen entropy $E_{e}$ that is defined as:

(10) $$E_{e}=-e_{1}\ln(e_{1})-e_{2}\ln(e_{2})-e_{3}\ln(e_{3})$$

where the $e_{j}=\lambda_{j}/\sum \lambda_{j}$ for j∈{1,2,3} represents the normalized eigenvalues summing up to 1. The optimal neighborhood radius $r_{\mathrm{opt}}$ is obtained according to the minimum criterion of eigen entropy $E_{e}$.

#### 2.4.2. ACFPFH Feature Description

The ACFPFH feature descriptor consisting of a 3-dimensional HSV color feature and the 33-dimensional FPFH geometric feature is proposed in this section, which is shown as Equation (11):

(11) $$\mathrm{ACFPFH}({\mathrm{PF}}_{i})=\mathrm{HSV}({\mathrm{PF}}_{i})+\mathrm{FPFH}({\mathrm{PF}}_{i})$$

where the PF_i_ is the key points of the pallet point cloud. The specific calculation procedures of color feature and geometric feature are as follows:

(1) Color feature calculation

The point cloud data acquired by the Percipio FM851-E2 3D vision sensor contain information such as the color and coordinates of the object. Due to the high correlation between components in RGB color space, color cognitive properties cannot be intuitively expressed. Therefore, RGB color space is not suitable for feature similarity detection. Compared with RGB color space, HSV color space is easier to distinguish and more consistent with human visual characteristics. H represents the hue, S represents the saturation and V represents the value. HSV color space is exploited to form a color feature descriptor of the key point ${\mathrm{PF}}_{i}$, and it can be converted from the RGB color space [31].

(12) $$\begin{array}{l} V=\max(R,G,B)\\ S=\left\{ \begin{array}{l} 0,\;\mathrm{if}\;V=0\\ \frac{\max(R,G,B)-\min(R,G,B)}{\max(R,G,B)} \end{array}\right.,\;\mathrm{otherwise}\\ H=\left\{ \begin{array}{l} 0,\;\mathrm{if}\;S=0\\ 60\times \left(G-B\right)/(S\times V),\;\mathrm{if}\;S\neq 0\;\mathrm{and}\;V=R\\ 60\times (2+\left(B-R\right)/\left(S\times V\right)),\;\mathrm{if}\;S\neq 0\;\mathrm{and}\;V=G\\ 60\times (4+\left(R-G\right)/\left(S\times V\right)),\;\mathrm{otherwise} \end{array}\right.\\ \mathrm{if}\;H<0,\;H=H+360 \end{array}$$

where the value range of R, G and B is [0,255], and the value ranges of H, S and V are [0,360], [0,1] and [0,255], respectively.

(2) Geometric feature calculation

**Figure 4:**
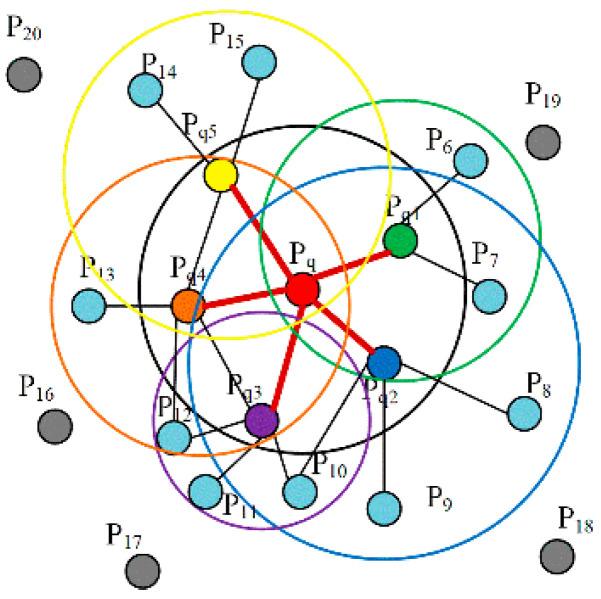
Neighborhood influence area of the point P_q_ for ACFPFH.

FPFH is an efficient local feature descriptor which reflects the normal relationship between query points and neighborhood points of point cloud data. The detailed calculation procedures are explained as follows:

Step 1: For each key point ${\mathrm{PF}}_{i}$ (or query point $P_{q}$), select all of the neighborhood points $P_{\mathrm{qj}}$ of the query point $P_{q}$ that are enclosed in the sphere with an adaptive optimal neighborhood $r_{\mathrm{opt}}$, as shown in Figure 4. The red point P_q_ in the middle of the figure is the query point, and the colored points P_q1_-P_q5_ in the black circle are the neighborhood points of P_q_, and those blue points P_q6_-P_q15_ are the neighborhood points of the colored points P_q1_–P_q5_.

**Figure 5:**
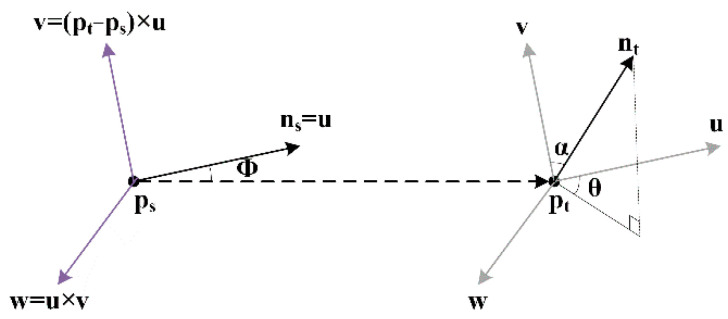
Local coordinate system.

Step 2: The point pairs $p_{s},p_{t}$ are generated based on the query point $P_{q}$ and the neighborhood points $P_{\mathrm{qj}}$. Estimate their corresponding normal $n_{s}$ and $n_{t}$. The relative relationship between the point pairs $p_{s},p_{t}$ is obtained by establishing a local frame, as shown in Figure 5.

Taking the point $p_{s}$ as the coordinate origin, the coordinate frame is set up with u, v and w axes. The axis is defined as:

(13) $$\begin{array}{l} u=n_{s}\\ v=(p_{t}-p_{s})\times u\\ w=u\times v \end{array}$$

Step 3: The angles α, φ and θ are calculated for representing the deviation between the normal vectors $n_{s}$ and $n_{t}$, which forms the simplified point feature histograms (SPFH).

(14) $$\begin{array}{l} \alpha =\arccos(\frac{v\cdot n_{t}}{\left|v\right|\left|n_{t}\right|})\\ \varphi =\arccos(\frac{u\cdot p}{\left|u\right|\left|p\right|})\\ \theta =\arctan(w\cdot n_{t},u\cdot n_{t}) \end{array}$$

where p represents $\frac{\left(p_{t}-p_{s}\right)}{{\left\|p_{t}-p_{s}\right\|}_{2}}$.

**Figure 6:**
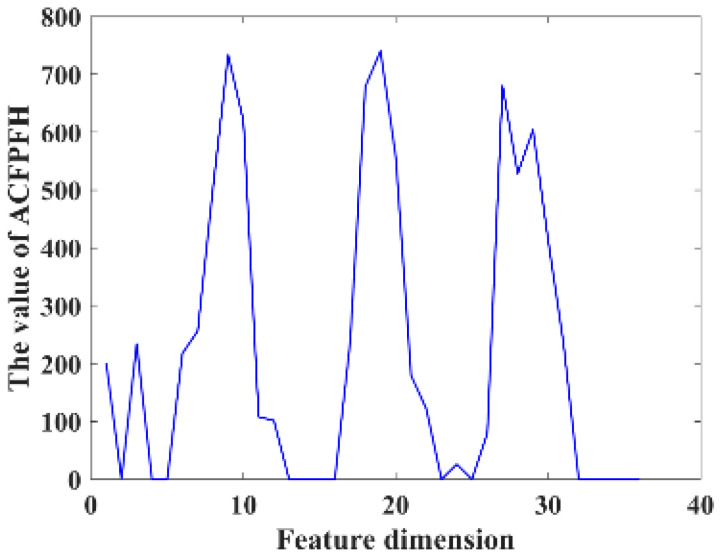
The ACFPFH of one point.

Step 4: For each neighborhood point $P_{\mathrm{qj}}$ of ${\mathrm{PF}}_{i}$, the $r_{\mathrm{opt}}$ is re-determined and the neighboring SPFH value is used to weight the final histograms of ${\mathrm{PF}}_{i}$, whose results are called FPFH.

(15) $$\mathrm{FPFH}({\mathrm{PF}}_{i})=\mathrm{SPFH}({\mathrm{PF}}_{i})+\frac{1}{k}\sum_{i=1}^{k}\frac{1}{\omega }\mathrm{SPFH}(P_{\mathrm{qj}})$$

where k represents the number of the neighborhood point P_qj_, and ω represents the weight, which is the reciprocal of the distance between P_s_ and P_t_. Figure 6 shows an example of the ACFPFH of one point.

### 2.5. Surface Matching

**Figure 7:**
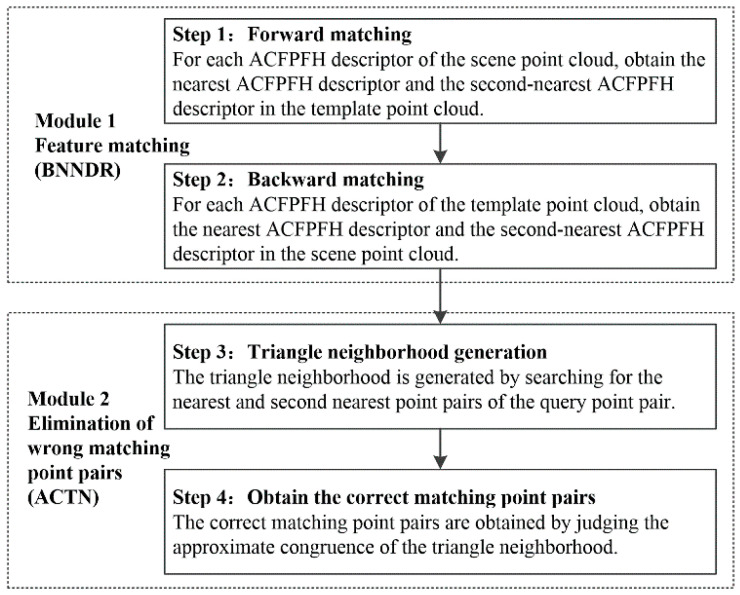
The flowchart the proposed surface matching method.

Accurate surface matching is an important prerequisite for point cloud registration, which directly affects the performance of pallet detection. For the traditional surface matching method, the one-way feature matching is performed and the method for eliminating incorrect matching point pairs only considers the relationship between points, leading to too many incorrect matching pairs. Therefore, a new surface matching method called BNNDR-ACTN is proposed, which includes feature matching based on the Bidirectional Nearest Neighbor Distance Ratio (BNNDR) and the incorrect matching point pairs’ elimination based on the Approximate Congruent Triangle Neighborhood (ACTN). The architecture of the proposed surface matching method is shown in Figure 7, and the detailed procedures are described as follows.

Module 1: Feature matching

The purpose of point cloud feature matching is to establish the relationship between the feature descriptors of the template point cloud and the scene point cloud, thereby obtaining the initial matching point pair.

Step 1: Forward matching

Define $F_{M}=\{f_{M}^{i}\}$ and $F_{S}=\{f_{S}^{j}\}$ as the sets of ACFPFH descriptors of the pallet template point cloud $Q_{M}$ and the scene point cloud $Q_{S}$, respectively. For each ACFPFH descriptor $f_{S}^{j}$ belonging to the scene point cloud, obtain the nearest ACFPFH descriptor $f_{M}^{i}$ and the second-nearest ACFPFH descriptor $f_{M}^{i}{}^{\prime }$ in the template point cloud, and their Euclidean distances are represented by $d(f_{S}^{j},f_{M}^{i})$ and $d(f_{S}^{j},{f_{M}^{i}}^{\prime })$. If the ratio of their distances satisfies the Equation (16), $\left(PF_{S}^{j},PF_{M}^{i}\right)$ can be considered as a candidate matching key point pair. That is, the key point $PF_{S}^{j}$ corresponds to $f_{S}^{j}$, and the key point $PF_{M}^{i}$ corresponds to $f_{M}^{i}$.

(16) $$\frac{d(f_{S}^{j},f_{M}^{i})}{d(f_{S}^{j},{f_{M}^{i}}^{\prime })}<th$$

where the threshold th is a constant between 0 and 1.

Step 2: Backward matching

For each ACFPFH descriptor $f_{M}^{i}$ belonging to the template point cloud, the nearest ACFPFH descriptor $f_{S}^{j}{}^{\prime }$ and the second-nearest ACFPFH descriptor $f_{S}^{j\prime \prime }$ are obtained in the scene point cloud, and their Euclidean distances are represented by $d(f_{M}^{i},f_{S}^{j\prime })$ and $d(f_{M}^{i},f_{S}^{j\prime \prime })$. If the ratio of their distances satisfies the Equation (17), $\left(PF_{S}^{j},PF_{M}^{i}\right)$ is a matching key point pair; otherwise, it is not a matching key point pair.

(17) $$\frac{d(f_{M}^{i},f_{S}^{j\prime })}{d(f_{M}^{i},f_{S}^{j\prime \prime })}<th$$

If the key points $PF_{S}^{j}$ and $PF_{S}^{j\prime }$ are the same point, $\left(PF_{S}^{j},PF_{M}^{i}\right)$ can be considered as a matching key point pair. The final matching key point pairs set $MP=\{Q_{S}^{i},Q_{M}^{i}\}$ is obtained by repeating the above steps.

Module 2: Elimination of wrong matching point pairs

The surface of the object is rough and noisy, which leads to some mismatching point pairs. Therefore, after obtaining initial matching pairs, the next step is to eliminate the wrong matching point pairs.

Step 3: Triangle neighborhood generation

Select a query point pair $\left(Q_{S}^{1},Q_{M}^{1}\right)$ from the matching key point pairs set $MP=\{Q_{S}^{j},Q_{M}^{i}\}$ and search for the nearest point pairs $\left(Q_{S}^{2},Q_{M}^{2}\right)$ and the second nearest point pairs $\left(Q_{S}^{3},Q_{M}^{3}\right)$, which can generate triangle neighborhood $T_{S}=(Q_{S}^{1},Q_{S}^{2},Q_{S}^{3})$ and $T_{M}=(Q_{M}^{1},Q_{M}^{2},Q_{M}^{3})$.

Step 4: Obtain the correct matching point pairs

The point pair $\left(Q_{S}^{1},Q_{M}^{1}\right)$ is considered as a correct matching point pair if the two triangles $T_{S}=(Q_{S}^{1},Q_{S}^{2},Q_{S}^{3})$ and $T_{M}=(Q_{M}^{1},Q_{M}^{2},Q_{M}^{3})$ are approximately congruent; otherwise, it will be regarded as a wrong matching point pair and should be eliminated. The Equation (18) is used to determine whether the two triangles are approximately congruent.

(18) $$\left\{ \begin{array}{l} -t<\frac{\mathrm{dist}(Q_{M}^{1},Q_{M}^{2})-\mathrm{dist}(Q_{S}^{1},Q_{S}^{2})}{W}<t\\ -t<\frac{\mathrm{dist}(Q_{M}^{1},Q_{M}^{3})-\mathrm{dist}(Q_{S}^{1},Q_{S}^{3})}{W}<t\\ -t<\frac{\mathrm{dist}(Q_{M}^{2},Q_{M}^{3})-\mathrm{dist}(Q_{S}^{2},Q_{S}^{3})}{W}<t \end{array}\right.$$

where dist( · ) represents the distance between two points, W=max(dist), and t represents the degree of approximation between the point pairs. Then, the final correct matching point pairs set $CP=\{Q_{CS}^{i},Q_{CM}^{i}\}$ is obtained by repeating the above steps.

Considering the stability of the triangle, each point in the point cloud is expanded into a triangular neighborhood. Therefore, the point-to-point matching problem is transformed into the neighborhood matching problem, which can obtain more feature information and improve registration accuracy. In addition, each point in the point cloud is regarded as the vertex of the triangle, which benefits the maintenance of the geometric characteristics of the original point cloud.

### 2.6. Point Cloud Registration

#### 2.6.1. Coarse Registration

The main task of point cloud coarse registration is to obtain the relationship between the template point cloud $Q_{M}$ and scene point cloud $Q_{S}$ and provide an ideal initial position for fine registration. This computation is based on the correct matching point pairs set $CP=\{Q_{CS}^{i},Q_{CM}^{i}\}$. The main steps are as follows:

Step 1: Three correspondences are randomly selected to estimate the rigid transformation matrix $R_{0}$ and $T_{0}$.

Step 2: Calculate the distance $D(R_{0},T_{0})$ between the point $Q_{CS}^{i}$ and the transformed point $Q_{TCM}^{i}$ based on the transformation matrix $R_{0}$ and $T_{0}$. Take the point in point set $Q_{CS}^{}$ whose corresponding distance $D(R_{0},T_{0})$ is less than threshold d_0_ as the inlier point; otherwise, consider it as an exterior point.

(19) $$D(R_{0},T_{0})=\left\|Q_{CS}^{i}-(R_{0}\times Q_{CM}^{i}+T_{0})\right\|$$

Step 3: Repeat the above steps to obtain a different rigid transformation matrix and count its corresponding number of inliers point until the maximum iteration number I_0_ is reached.

Step 4: Obtain the final rigid transformation matrix ${R_{0}}^{\prime }$ and ${T_{0}}^{\prime }$ with the most interior points; the template point cloud $Q_{M}$ is transformed into the coordinate system of the scene point cloud $Q_{S}$ to complete the coarse registration. Define the transformed template point cloud $Q_{M}$ as $Q_{\mathrm{MT}}$.

#### 2.6.2. Fine Registration

The Iterative Closest Point (ICP) algorithm is used to achieve point cloud fine registration. It is based on minimizing the error function to calculate the optimal rotation matrix and translation matrix. The specific procedures of fine registration are as follows:

Step 1: For each point $Q_{MT}^{i}$ in the transformed template point cloud $Q_{MT}$, search for its nearest neighbor point $Q_{S}^{i}$ in the scene point cloud $Q_{S}$, thereby generating the corresponding points pairs set $C_{F}=\{Q_{MT}^{i},Q_{S}^{i}\}$.

Step 2: Use the least square method to solve the rotation matrix $R_{n}$ and translation matrix $T_{n}$ with the smallest average distance $e_{n}$ between the corresponding points.

(20) $$e_{n}=\frac{1}{k}\sum_{i=1}^{k}{\left\|Q_{S}^{i}-(Q_{MT}^{i}\times R_{n}+T_{n})\right\|}^{2}$$

where n is the number of iterations and k is the number of the corresponding points.

Step 3: Repeat the above steps and obtain the optimal rotation matrix $R_{f}$ and translation matrix $T_{f}$ until $e_{n}$ is smaller than distance threshold $e_{f}$, or the maximum number of iterations $I_{f}$ is reached. The new template point cloud $Q_{\mathrm{MF}}$ is obtained by using the transformation matrix $R_{f}$ and $T_{f}$, and fine registration is completed.

## 3. Case Studies

### 3.1. Evaluation Index

In order to validate the performance of the proposed ACFPFH feature descriptor and the overall registration method, two representative indicators are developed to evaluate the experiment results, and the details of these indicators are briefly described as follows. The experiment was performed using MATLAB Code on a desktop with 3.6 GHz inter^®^ Core™ i7-11700kf CPU and 16 G memory.

(1) Precision-recall curve

The precision–recall curve (PRC) is used to evaluate the descriptiveness of a feature descriptor. The precision is calculated as the number of correct matching point pairs with respect to the total number of matching point pairs:

(21) $$\mathrm{Precision}=\frac{N_{\mathrm{CP}}}{N_{\mathrm{MP}}}$$

where $N_{\mathrm{CP}}$ represents the number of correct matching point pairs, and $N_{\mathrm{MP}}$ represents the number of matching point pairs.

The recall is calculated as the number of correct matching point pairs with respect to the number of key points of the template point cloud:

(22) $$\mathrm{Recall}=\frac{N_{\mathrm{CP}}}{N_{\mathrm{PF}}}$$

where $N_{\mathrm{PF}}$ represents the number of key points of the template point cloud. The value of the threshold th used for performing feature matching in Section 2.5 varies from 0 to 1 to calculate the precision and recall under each threshold and obtain the PRC.

(2) Root mean square error

Root mean square error (RMSE) is the error evaluation index commonly used in point cloud registration, which represents the average of the sum of squared distances between the corresponding points of the two point clouds. It is defined as:

(23) $$\mathrm{RMSE}=\sqrt{\frac{\sum_{i=1}^{m}\left||P_{i}-Q_{j}|\right|}{m}}$$

where $P_{i}$ and $Q_{j}$ are the corresponding points, and m is the number of the corresponding point pairs. The smaller the value of RMSE, the better the fine registration result.

### 3.2. Experiment Preparation

**Figure 8:**
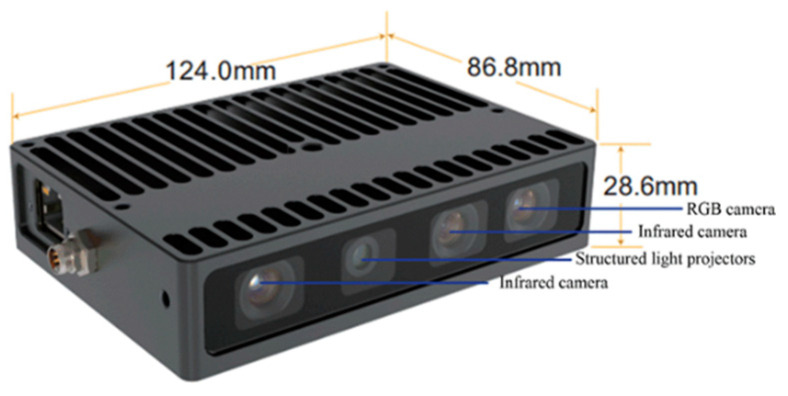
Structure of Percipio FM851-E2.

In order to verify the effectiveness and feasibility of the proposed pallet detection method, a widely used industrial camera called the Percipio FM851-E2 3D vision sensor is adopted to acquire point cloud data for comparative analysis of the results. The vision sensor shown in Figure 8 consists of an RGB camera and a depth sensor which is composed of an infrared camera and structured light projector. Its length, width and height are 124.0 mm, 86.8 mm and 28.6 mm, respectively. The RGB camera captures the RGB image with a resolution of 1280 × 960 and the depth sensor captures the depth image with a resolution of 320 × 240. Percipio FM851-E2 3D vision sensor active binocular vision technology is used for measuring the distance, and its operative range is from 0.7 m to 6.0 m.

**Figure 9:**
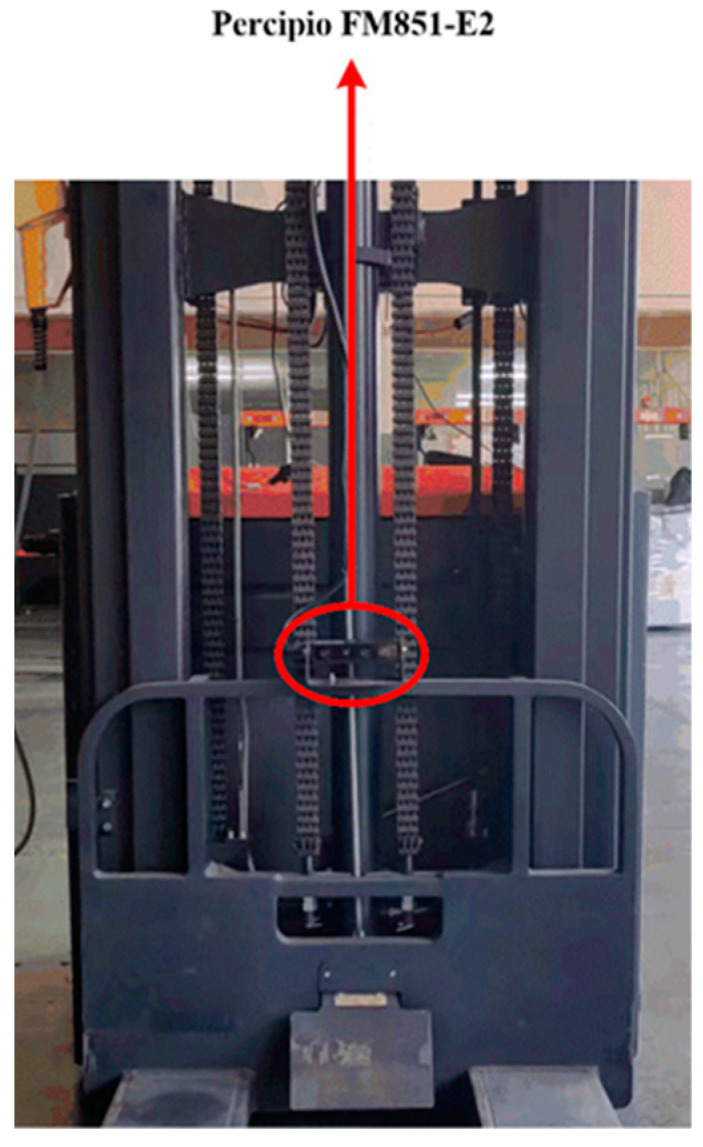
The installation position of the Percipio FM851-E2.

**Figure 10:**
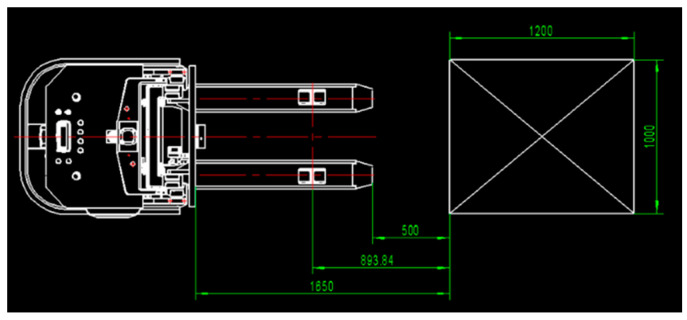
The pose relationship between the 3D vision sensor and the pallet.

The Percipio FM851-E2 3D vision sensor is mounted on the top of the carriage of a real automated guided vehicle, which means the camera will move along with the forks, as shown in Figure 9. Given that the length of the fork is 1150 mm, the distance between the top of the fork and the front face of the pallet is set to 500 mm so that the automated guided vehicle is able to adjust its position. Meanwhile, it is necessary to ensure that the fork is perpendicular to the front face of the pallet and that the center of the sensor is in line with the center of the pallet. The specific placement of the pallet is shown in Figure 9 and Figure 10. Considering the effect of illumination on point cloud data acquired by the sensor, all experiments are carried out under normal daytime illumination. In this case, the size of the pallet is 1200 mm × 1000 mm × 150 mm, and it is extracted from the scene point cloud and considered as the template point cloud.

### 3.3. Case Study I

#### 3.3.1. Implementation Process

**Figure 11:**
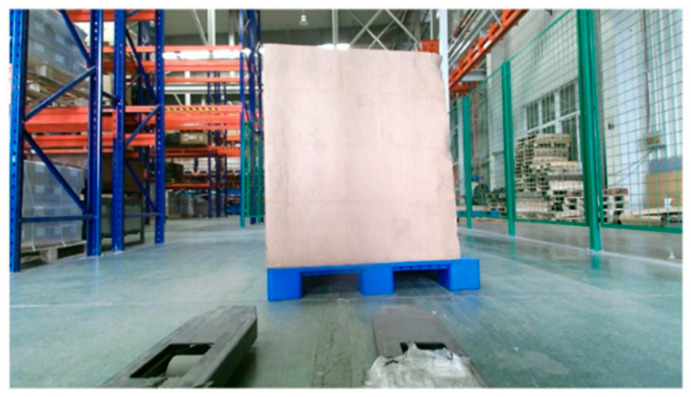
Color image of the original ground scene.

**Figure 12:**
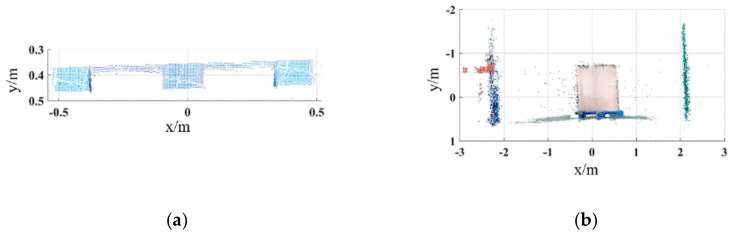
Pallet template point cloud and original ground scene point cloud. (**a**) Pallet template point cloud. (**b**) Original ground scene point cloud.

**Figure 13:**
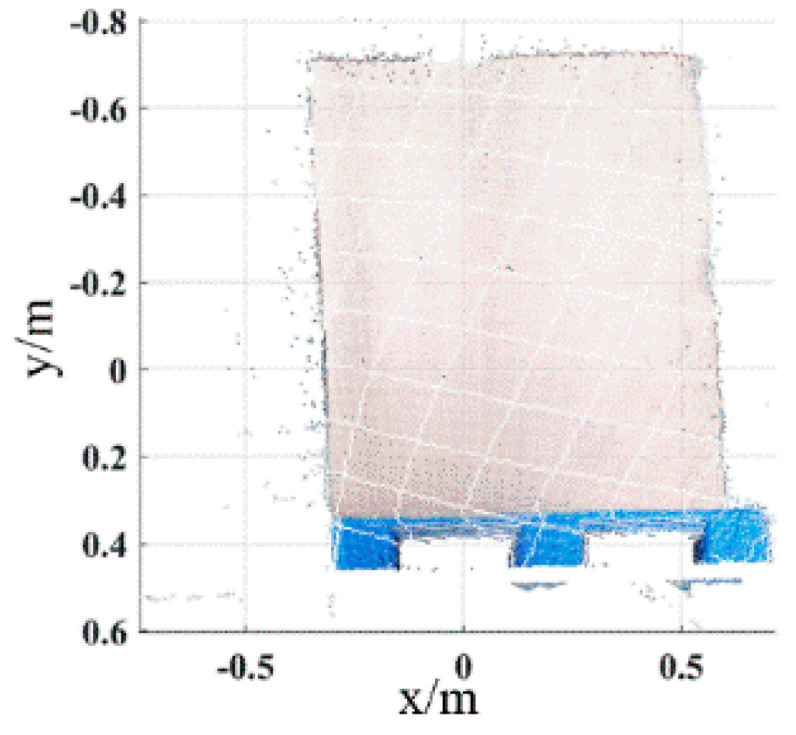
Pallet template point cloud and original ground scene point cloud.

**Figure 14:**
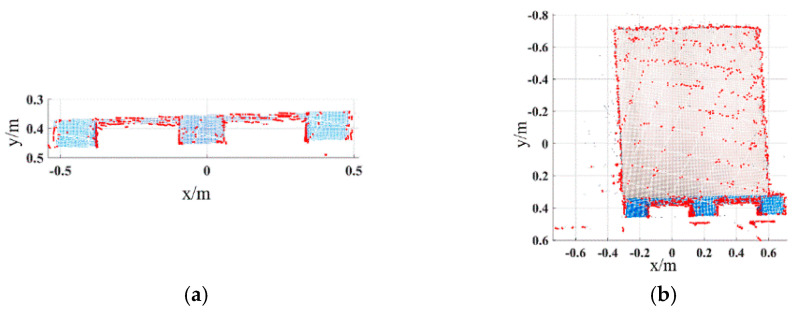
Key points of pallet template point cloud and ground scene point cloud. (**a**) Key points of pallet template point cloud. (**b**) Key points of ground scene point cloud.

For the pallets on the ground, the color image of the scene is acquired by the Percipio FM851-E2 3D vision sensor at the same distance of 500 mm, which is shown in Figure 11. Figure 12 shows the pallet template point cloud and the scene point cloud. Then, the outliers of the scene point cloud are eliminated. The normal of the ground and the wall are [0, 1, 0] and [0, 0, 1], respectively. Set distance threshold $D_{\varepsilon }=0.02\;m$ and angle threshold $\beta_{\varepsilon }=5{}^{\circ}$; the plane segmentation is performed for the scene point cloud after removing outliers, and the result is shown in Figure 13. The ISS algorithm is used to extract key points with the search radius of 0.013 m and thresholds $\kappa_{1}=0.6,\kappa_{2}=0.75$, which can guarantee the efficiency and accuracy of the method. The number of points in the pallet template point cloud decreased from 2661 to 492, and the number of points in the scene point cloud decreased from 41,351 to 576, as shown in Figure 14, and the red points in Figure 14 represent the key points. The point cloud image shown below contains the RBG information of the point cloud and therefore has different colors.

**Figure 15:**
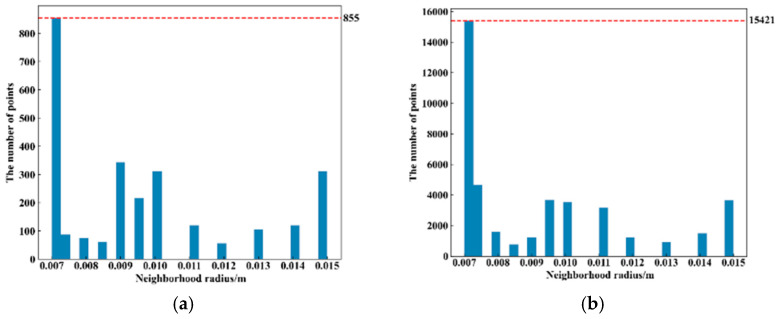
Adaptive optimal neighborhood radius distribution of point cloud. (**a**) Adaptive optimal neighborhood radius distribution of template point cloud. (**b**) Adaptive optimal neighborhood radius distribution of ground scene point cloud.

It is necessary to determine the adaptive neighborhood radius for each point before calculating the ACFPFH feature descriptor. Given that the interval between two sampling points of point cloud data acquired by Percipio FM851-E2 3D vision sensor is 0.007 m, set the radius range $r_{\min}=0.007m$, $r_{\max}=0.015m$, $r_{mid}=0.011m$ and $r_{\Delta }=0.0005m$. The adaptive optimal neighborhood radius of each point is obtained with the minimum criterion of neighborhood information entropy function. The adaptive optimal neighborhood radius distribution of pallet template point cloud and scene point cloud is shown in Figure 15. The horizontal axis represents the value of the neighborhood radius, and the vertical axis represents the number of points corresponding to each neighborhood radius. Among the 2661 points in the pallet template point cloud, there are 855 points with an optimal neighborhood radius of 0.007 m. Among the 41,351 points in the ground scene point cloud, there are 15,421 points with an optimal neighborhood radius of 0.007 m. It meets where the optimal neighborhood radius of points is concentrated at the given minimum neighborhood radius, which aids in improving the efficiency.

**Figure 16:**
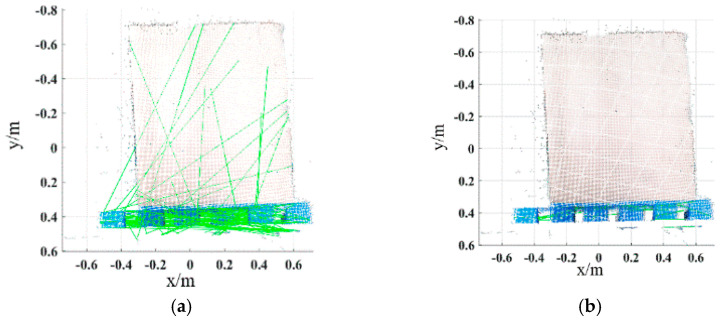
The result of feature matching. Schemes follow another format. (**a**) Feature matching. (**b**) Elimination of wrong matching point pairs. The green lines are the lines connecting the matching point pairs.

Extract the HSV color components of the key points of the pallet template point cloud and the scene point cloud, and calculate the geometric feature based on the adaptive optimal neighborhood radius. The ACFPFH feature descriptor is obtained by superimposing the color and geometric features. The feature matching is completed with the distance ratio threshold of th=0.75. The initial matching result is shown in Figure 16a. The green line connects the corresponding points between the pallet template point cloud and the scene point cloud. Obviously, there are some wrong matching point pairs. The wrong matching point pairs are eliminated by using the wrong matching point pairs elimination algorithm based on the ACTN, and the result is shown in Figure 16b. The RANSAC algorithm is used for coarse registration to calculate the rough transformation matrix, and the ICP algorithm is used to obtain a final transformation matrix and complete fine registration. The parameters of the final transformation matrix are as follows:

(24) $$R=\left[\begin{array}{ccc} 0.9889 & 0.0067 & -0.1487\\ -0.0099 & 0.9997 & -0.0206\\ 0.1485 & 0.0219 & 0.9887 \end{array}\right]T=\left[\begin{array}{l} 0.4709\\ 0.0312\\ 0.0723 \end{array}\right]$$

#### 3.3.2. Performance Evaluation

The PRC is used to evaluate the descriptiveness of a feature descriptor. The ACFPFH feature descriptor is compared with the classical feature descriptors including FPFH, CFPFH and Signature of Histogram of Orientation (SHOT) with the fixed neighborhood radius.

**Figure 17:**
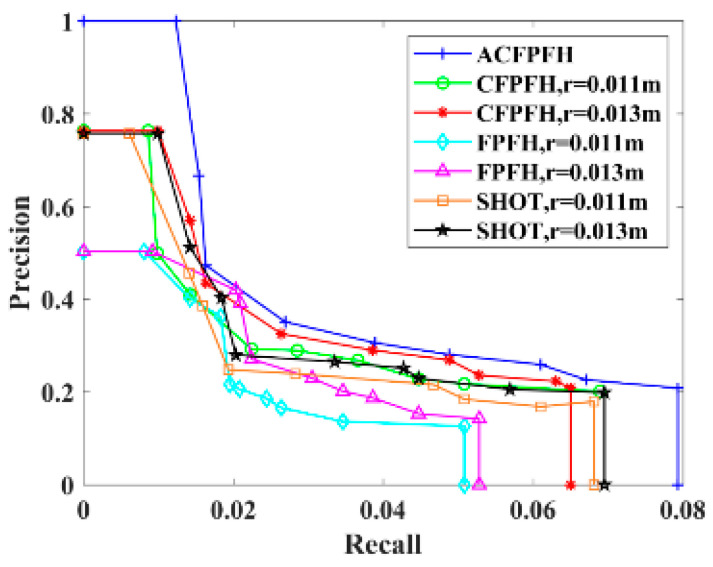
PRC of different feature descriptors.

The set th={0.2,0.4,0.6,0.75,0.85,0.925,0.95,9.975,1.0} is considered as the selected distance ratio threshold set of the feature matching stage, and the PRC corresponding to different feature descriptors is obtained, as shown in Figure 17. Take th = 0.75 to compare the accuracy of different feature descriptors, as shown in Table 2. Table 3 lists the time required for feature extraction of the scene point cloud of different feature descriptors, and the bold characters are the experimental results of the proposed method.

Traditional feature descriptors such as SHOT and FPFH only describe the geometric feature of the pallet and ignore the color information, so the precision is lower. The CFPFH feature descriptor considers the HSV color information, which improves the precision. The neighborhood radiuses of the above three feature descriptors are obtained by complex and inefficient manual debugging methods, which are not suitable for all points in the point cloud. A large neighborhood radius leads to too many key points in the neighborhood, which reduces the speed of feature extraction. The ACFPFH feature descriptor not only contains color information but also adaptively selects the optimal neighborhood radius for each key point, so that it performs better in terms of effectiveness and precision.

It is well known that the closer the curve is to the upper right, the better the performance of the feature descriptor in the PRC graph. It can be seen from Figure 17 that comparing with SHOT, FPFH and CFPFH feature descriptors with fixed radiuses, the ACFPFH feature descriptor has the best performance. It can be seen from Table 2 and Table 3 that when th = 0.75, compared with the SHOT feature descriptor with a neighborhood radius of 0.011 m, the precision is improved by 29.40%, and the time required for feature extraction is reduced by 14.57%. Compared with the FPFH feature descriptor with a neighborhood radius of 0.011 m, the precision is improved by 39.10%, and the time required for feature extraction is reduced by 11.03%. Compared with the CFPFH feature descriptor with a neighborhood radius of 0.011 m, the precision is improved by 16.68%, and the feature extraction time is reduced by 18.87%.

**Figure 18:**
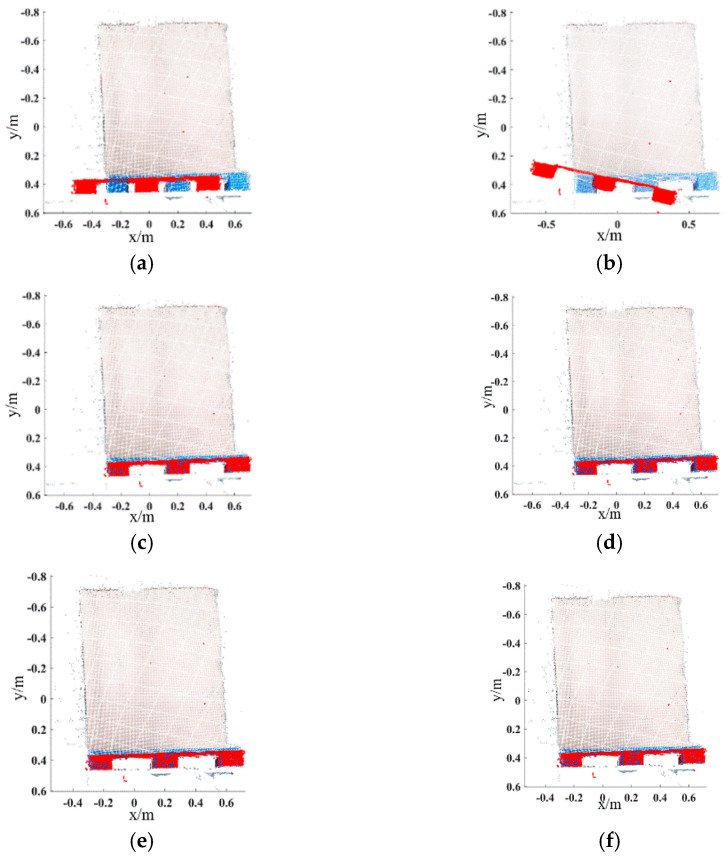
Point registration results based on different methods in the ground scene. (**a**) Initial position. (**b**) Traditional ICP. (**c**) The method based on SHOT + ICP. (**d**) The method based on FPFH + ICP. (**e**) The method based on CFPFH + ICP. (**f**) The proposed method.

The RMSE and runtime are used to evaluate the performance of the registration algorithms. Popular algorithms including ICP, SHOT + ICP, FPFH + ICP and CFPFH + ICP are selected to compare with the proposed method in this paper. The number of iterations, the RMSE and the runtime of the above methods are detailed in Table 4. The initial position relationship of the pallet template point cloud and the scene point cloud and the registration results of different methods are shown in Figure 18, and the red points are the template point cloud.

The following conclusions can be drawn from Table 4 and Figure 18: The traditional ICP algorithm has a large registration error due to the large initial pose difference. It takes 27.256 s to realize registration, which cannot meet the real-time requirements in intelligent manufacturing systems. The modified ICP registration methods such as SHOT + ICP, FPFH + ICP and CFPFH + ICP perform coarse registration, providing a better initial position for the fine registration by the ICP algorithm. Compared with the traditional ICP algorithm, the RMSE and runtime are reduced. However, the feature descriptors used by the above methods lack neighborhood selection criteria, which leads to an increase in the overall registration runtime. The proposed method has minimal registration error and the least runtime, which shows higher efficiency and proves that the proposed method has a more significant improvement than other methods. Furthermore, the precision and efficiency of the proposed method also meet the production requirement in intelligent manufacturing systems.

### 3.4. Case Study II

**Figure 19:**
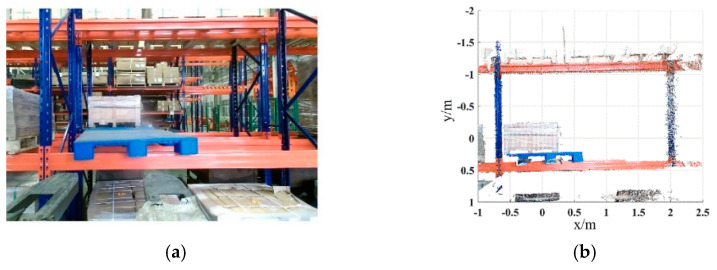
Shelf scene. (**a**) Color image. (**b**) Point cloud.

**Figure 20:**
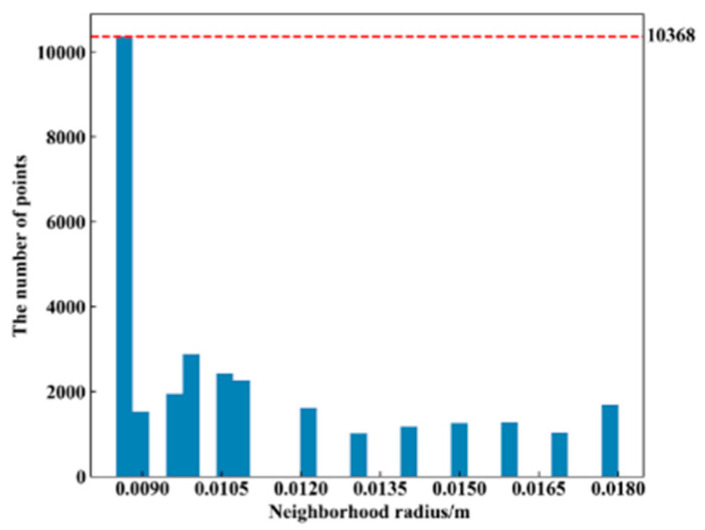
Adaptive optimal neighborhood radius distribution of point cloud in the shelf scene.

**Figure 21:**
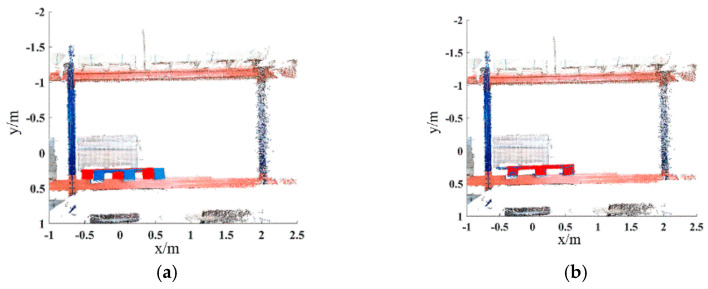
The result of point registration in the shelf scene. (**a**) Initial pose relationship. (**b**) Fine registration. The red points represents the template point cloud.

Shelves are widely used in intelligent manufacturing systems, which can improve the utilization rate of warehouse space and realize the rational allocation of resources while ensuring the quality of goods. Hence, it is necessary to complete the pallet detection of the shelf scene. The color image and the point cloud of the shelf scene are acquired with the same distance from the ground scene, as shown in Figure 19. The same pallet template point cloud is used to perform pallet detection of the shelf scene, and the parameters are consistent with the ground scene in Case Study I. After extracting the key points, the number of points in the scene point cloud decreased from 30,469 to 658. The adaptive optimal neighborhood radius distribution of the scene point cloud in the shelf scene is shown in Figure 20. Figure 21 and Table 5 show the registration result.

Compared with the traditional ICP algorithm, the RMSE of the proposed method is greatly reduced, and the runtime is reduced from 29.523 s to 0.989 s, which proves that the efficiency and accuracy have been greatly improved. Compared with other modified ICP registration methods, the RMSE of the proposed method is the smallest, and the runtime is the shortest. In summary, the proposed method still achieves optimal performance in the shelf scene, which shows its effectiveness in different scenes. Furthermore, the above case studies demonstrate that the proposed method can be well applied in intelligent manufacturing systems to realize accurate and efficient pallet detection. In addition, feature descriptors can often determine the final performance in the process of point cloud registration. Combining a good feature descriptor with a good matching strategy would improve the efficiency of point cloud registration.

## 4. Conclusions

A novel pallet detection method for automated guided vehicles based on point cloud data is proposed in this paper. The contributions of this paper can be concluded as follows:

1. A novel pallet detection method for automated guided vehicles based on point cloud data is proposed, which can be used for automated guided vehicles to perform automated and effective pallet handling, thereby promoting the transformation and upgrading of the manufacturing industry.
2. A new Adaptive Color Fast Point Feature Histogram (ACFPFH) feature descriptor has been built for the description of pallet features, which overcomes shortcomings such as low efficiency, time-consumption, poor robustness, and random parameter selection in feature description.
3. A new surface matching method called the Bidirectional Nearest Neighbor Distance Ratio-Approximate Congruent Triangle Neighborhood (BNNDR-ACTN) is proposed, which transforms the point-to-point matching problem into the neighborhood matching problem and can obtain more feature information and improve the detection accuracy.

Due to the measurement accuracy of the 3D vision sensor being easily affected by environmental factors such as illumination and obstacles, a more robust and efficient pallet detection method will be researched, which is suitable for more complex scenarios.

## Figures and Tables

**Table 1:** Dimensional features judgment of local neighborhood of point cloud.

Eigenvalue Relation	Dimensionality Feature	Eigenvalue Relation
$\lambda_{1}\gg \lambda_{2}\approx \lambda_{3}$	Linearity feature	$\lambda_{1}\gg \lambda_{2}\approx \lambda_{3}$
$\lambda_{1}\approx \lambda_{2}\gg \lambda_{3}$	Planarity feature	$\lambda_{1}\approx \lambda_{2}\gg \lambda_{3}$
$\lambda_{1}\approx \lambda_{2}\approx \lambda_{3}$	Scattering feature	$\lambda_{1}\approx \lambda_{2}\approx \lambda_{3}$

**Table 2:** Precision and recall comparison of different feature descriptors.

Name	Feature Dimension	Neighborhood Radius/m	Recall	Precision	Accuracy Comparison of ACFPFH with Other Feature Descriptors (%)
SHOT	352	0.011	0.0193	0.2481	29.40
		0.013	0.0203	0.2798	20.38
FPFH	33	0.011	0.0183	0.2140 0.2712	39.10
		0.013	0.0224		22.82
CFPFH	36	0.011	0.0219	0.2928	16.68
		0.013	0.0264	0.3256	7.34
**ACFPFH**	**36**	**Adaptive**	**0.0269**	**0.3514**	/

**Table 3:** Feature extraction time comparison of different feature descriptors.

Name	Feature Dimension	Neighborhood Radius/m	Feature Extraction Time/s	Time Comparison of ACFPFH with Other Feature Descriptors (%)
SHOT	352	0.011	0.151	14.57
		0.013	0.185	30.27
FPFH	33	0.011	0.145	11.03 25.86
		0.013	0.174	
CFPFH	36	0.011	0.159	18.87
		0.013	0.193	33.16
**ACFPFH**	**36**	**Adaptive**	**0.129**	/

**Table 4:** RMSE and elapsed time comparison of different feature descriptors.

Method	The Number of Iterations	RMSE	The Runtime/s
Traditional ICP	113	0.040344	27.256
SHOT + ICP	82	0.024791	0.986
FPFH + ICP	24	0.026589	0.948
CFPFH + ICP	44	0.021559	1.039
**ACFPFH**	**26**	**0.009251**	**0.853**

**Table 5:** RMSE and elapsed time comparison of different feature descriptors in the shelf scene.

Method	The Number of Iterations	RMSE	The Runtime/s
Traditional ICP	68	0.041553	29.523
SHOT + ICP	49	0.025987	1.174
FPFH + ICP	32	0.026751	1.118
CFPFH + ICP	36	0.018954	1.326
**ACFPFH**	**23**	**0.009032**	**0.989**

## Data Availability

The data presented in this study are available on request from the corresponding author.

## References

[1] Ambroz M. (2017). Raspberry Pi as a low-cost data acquisition system for human powered vehicles. Measurement.

[2] Li Z., Barenji A.V., Jiang J., Zhong R.Y., Xu G. (2018). A mechanism for scheduling multi robot intelligent warehouse system face with dynamic demand. J. Intell. Manuf..

[3] Casado F., Lapido Y.L., Losada D.P. (2017). Pose Estimation and Object Tracking Using 2D Images. Procedia Manuf..

[4] Sriram K.V., Havaldar R.H. (2021). Analytical review and study on object detection techniques in the image. Int. J. Modeling Simul. Sci. Comput..

[5] Wang Q., Tan Y., Mei Z. (2019). Computational Methods of Acquisition and Processing of 3D Point Cloud Data for Construction Applications. Arch. Comput. Methods Eng..

[6] Camurri M., Vezzani R., Cucchiara R. (2014). 3D Hough transform for sphere recognition on point clouds: A systematic study and a new method proposal. Mach. Vis. Appl..

[7] García-Pulido J.A., Pajares G., Dormido S., de la Cruz J.M. (2017). Recognition of a landing platform for unmanned aerial vehicles by using computer vision-based techniques. Expert Syst. Appl..

[8] Seidenari L., Serra G., Bagdanov A.D., Del Bimbo A. (2014). Local Pyramidal Descriptors for Image Recognition. IEEE Trans. Pattern Anal. Mach. Intell..

[9] Chen J., Chen L. (2021). Multi-Dimensional Color Image Recognition and Mining Based on Feature Mining Algorithm. Autom. Control. Comput. Sci..

[10] Joshi K.D., Chauhan V., Surgenor B. (2018). A flexible machine vision system for small part inspection based on a hybrid SVM/ANN approach. J. Intell. Manuf..

[11] Bastian B.T., Charangatt Victor J. (2020). Detection and pose estimation of auto-rickshaws from traffic images. Mach. Vis. Appl..

[12] Li J., Kang J., Chen Z., Cui F., Fan Z. (2020). A Workpiece Localization Method for Robotic De-Palletizing Based on Region Growing and PPHT. IEEE Access.

[13] Syu J.-L., Li H.-T., Chiang J.S. (2016). A computer vision assisted system for autonomous forklift vehicles in real factory environment. Multimed. Tools Appl..

[14] Li T., Huang B., Li C. (2019). Application of convolution neural network object detection algorithm in logistics warehouse. J. Eng..

[15] Shao Y., Wang K., Du S., Xi L. (2018). High definition metrology enabled three dimensional discontinuous surface filtering by extended tetrolet transform. J. Manuf. Syst..

[16] Huang D., Du S., Li G., Zhao C., Deng Y. (2018). Detection and monitoring of defects on three-dimensional curved surfaces based on high-density point cloud data. Precis. Eng..

[17] Jia S., Deng Y., Lv J., Du S., Xie Z. (2022). Joint distribution adaptation with diverse feature aggregation: A new transfer learning framework for bearing diagnosis across different machines. Measurement.

[18] He K., Zhang M., Zuo L., Alhwiti T., Megahed F.M. (2014). Enhancing the monitoring of 3D scanned manufactured parts through projections and spatiotemporal control charts. J. Intell. Manuf..

[19] Seelinger M., Yoder J.D. Automatic pallet engagment by a vision guided forklift. Proceedings of the IEEE International Conference on Robotics and Automation (ICRA).

[20] Lecking D., Wulf O., Wagner B. Variable pallet pick-up for automatic guided vehicles in industrial environments. Proceedings of the 11th IEEE International Conference on Emerging Technologies and Factory Automation.

[21] Guo Y., Bennamoun M., Sohel F., Lu M., Wan J. (2014). 3D Object Recognition in Cluttered Scenes with Local Surface Features: A Survey. IEEE Trans. Pattern Anal. Mach. Intell..

[22] Liu Y., Kong D., Zhao D., Gong X., Han G. (2018). A Point Cloud Registration Algorithm Based on Feature Extraction and Matching. Math. Probl. Eng..

[23] Tao Y., Zhou J. (2017). Automatic apple recognition based on the fusion of color and 3D feature for robotic fruit picking. Comput. Electron. Agric..

[24] Liu J., Bai D., Chen L. (2018). 3-D point cloud registration algorithm based on greedy projection triangulation. Appl. Sci..

[25] Li P., Wang J., Zhao Y., Wang Y., Yao Y. (2016). Improved algorithm for point cloud registration based on fast point feature histograms. J. Appl. Remote Sens..

[26] Kitt B., Geiger A., Lategahn H. Visual Odometry based on Stereo Image Sequences with RANSAC-based Outlier Rejection Scheme. Proceedings of the IEEE Intelligent Vehicles Symposium (IV), University of California, San Diego (UCSD).

[27] Xu G., Pang Y., Bai Z., Wang Y., Lu Z. (2021). A Fast Point Clouds Registration Algorithm for Laser Scanners. Appl. Sci..

[28] Napoli A., Glass S., Ward C., Tucker C., Obeid I. (2017). Performance analysis of a generalized motion capture system using microsoft kinect 2.0. Biomed. Signal Proces..

[29] Demantké J., Mallet C., David N., Vallet B. (2012). Dimensionality based scale selection in 3D lidar point clouds. Int. Arch. Photogramm..

[30] Weinmann M., Jutzi B., Mallet C. Semantic 3D scene interpretation: A framework combining optimal neighborhood size selection with relevant features. Proceedings of the ISPRS annals of the photogrammetry, remote sensing and spatial information sciences.

[31] Chernov V., Alander J., Bochko V. (2015). Integer-based accurate conversion between RGB and HSV color spaces. Comput. Electr. Eng..

